# Trends in the incidence of physician-diagnosed posttraumatic stress disorder among active-duty U.S. military personnel between 1999 and 2008

**DOI:** 10.1186/s40779-019-0198-5

**Published:** 2019-03-25

**Authors:** Kenneth L. Cameron, Rodney X. Sturdivant, Susan P. Baker

**Affiliations:** 1 John A. Feagin Jr. Sports Medicine Fellowship, Keller Army Hospital, United States Military Academy, West Point, New York, 10996 USA; 2 Department of Mathematics, Physics, and Statistics, Azusa Pacific University, Azusa, California USA; 3 Department of Health Policy and Management, Johns Hopkins Bloomberg School of Public Health, Baltimore, MD USA

**Keywords:** PTSD, Incidence rate, Trends, Military, OIF, OEF, Epidemiology

## Abstract

**Background:**

The impact of combat operations in Iraq and Afghanistan on the incidence of post-traumatic stress disorder (PTSD) in military service members has been poorly quantified. The purpose of this study was to examine trends in the incidence rate of physician-diagnosed PTSD in active-duty military personnel between 1999 and 2008.

**Methods:**

We conducted a retrospective cohort study utilizing data extracted from the Defense Medical Surveillance System to identify incident cases of PTSD within the study population. The incidence rate of physician-diagnosed PTSD was the primary outcome of interest. Multivariable Poisson regression was used to analyze the data.

**Results:**

The overall incidence rate of PTSD among all active-duty US military personnel was 3.84 (95% CI: 3.81, 3.87) cases per 1000 person-years. The adjusted average annual percentage increase in the incidence rate of PTSD prior to the initiation of Operation Iraqi Freedom (OIF) was a modest 5.02% (95% CI: 1.85, 8.29%). Following the initiation of OIF, the average annual percentage increase in the rate of PTSD was 43.03% (95% CI: 40.55, 45.56%). Compared to the baseline period between 1999 and 2002, the incidence rate of PTSD in 2008 was nearly 7 times higher (RR = 6.85, 95% CI: 6.49, 7.24). Significant increases in the incidence rate of PTSD were observed following the initiation of OIF regardless of sex, age, race, marital status, military rank, or branch of military service. Notably, the rate of PTSD among females was 6–7 times higher prior to OIF, but there was no difference by gender by 2008.

**Conclusions:**

Overall, these data quantify the significant increase in the incidence rate of PTSD following the initiation of combat operations in Iraq and Afghanistan within the active-duty military population during the study period.

## Background

Mental health conditions were common in military populations prior to the initiation of combat operations in Iraq (OIF) and Afghanistan (OEF) [1, 2]; however, they subsequently emerged as the “signature wounds” among US military personnel deployed in support of these operations [3]. Among these mental health conditions, posttraumatic stress disorder (PTSD) and major depression have received the greatest attention. A report by the RAND corporation suggested that approximately 300,000 US service members deployed prior to October 2007 suffered from PTSD. They estimated the per-case cost to range between $5904 and $10,298, with the total economic impact of PTSD and major depression reaching upwards of $6.2 billion [3]. Within military populations, PTSD has also been associated with high rates of attrition, absenteeism, occupational disability, impaired social functioning, and reduced health-related quality of life [1, 2, 4–8].

Several studies have documented the prevalence of PTSD within military and veteran populations, and estimates have varied widely [9–13]. Several factors have been described to explain these differences including variability between studies in methodology, target populations and sampling frame, response rates, operational case definitions for PTSD and measurement strategies [9–11]. The majority of published studies have reported estimates of point prevalence of PTSD and the majority have ranged between 5 and 12% [9]. A recent meta-analysis estimated the prevalence of PTSD in OEF and OIF veterans to be 23%; however, estimates in the literature ranged from 1.4 to 60.0% [13]. Measures of prevalence represent the number of new and existing cases in a population for a given period of time (period prevalence) or at a specific point in time (point prevalence). While estimates of prevalence provide useful information for the planning and the allocation of healthcare resources, the information they provide about the occurrence of new cases and the determinants of disease are limited [14]. In contrast, incidence rates specifically focus on the number of new cases of a mental health condition that occur during a specified period of time divided by person-time at risk for developing the condition in a population.

Few studies have examined the incidence of PTSD in active-duty military populations. Smith et al. [15] reported estimates of the cumulative incidence of PTSD in a prospective population-based military cohort. They indicated that the cumulative incidence of PTSD was the highest in service members who were deployed and reported combat exposure (7.6–8.7%) followed by nondeployers (2.3–3.0%) and deployers who did not report combat exposure (1.4–2.1%). They also noted that self-reported combat exposure, female sex, divorced marital status, and enlisted rank were associated with a higher incidence of PTSD in at least three of the four branches of the military service. While this study provides unique insight into the occurrence of new cases and the determinants of PTSD, it did not evaluate population-based trends in the incidence of PTSD over time. Furthermore, the population of this cohort may not be representative of the entire military population by design [9, 15]. The overall objective of the present investigation was to examine the incidence rate of physician-diagnosed PTSD among all US service members on active duty between 1999 and 2008. Our primary research hypothesis was that the incidence rate of PTSD would be relatively stable prior to the initiation of combat operations in Iraq and Afghanistan but would significantly increase after 2002. A secondary hypothesis was that demographic and occupational factors, primarily those examined by Smith et al. [15], would be associated with the incidence rate of PTSD during the study period.

## Methods

### Design and setting

A retrospective cohort study was conducted to examine the incidence rate and risk factors associated with the primary occurrence of PTSD among active-duty US service members between 1999 and 2008. The US military population represents an open and dynamic cohort at increased risk for PTSD since the initiation of combat operations in Iraq and Afghanistan. Using established methods described previously [16–18], data were extracted from the Defense Medical Surveillance System (DMSS), which serves as the central repository for all medical surveillance data and captures all healthcare encounters between providers and beneficiaries through the Military Health System for all four branches of military service [19, 20]. The structure, utility, and capabilities of the DMSS for public health surveillance and epidemiological research have been described in the literature [16, 19–21]. The DMSS has been utilized to examine the burden of mental health disorders within military populations previously [1, 2]. Data for hospitalizations and ambulatory visits are summarized in DMSS by major diagnostic categories using International Classification of Diseases, Ninth Revision, Clinical Modification (ICD-9-CM) codes to document every patient encounter occurring in military treatment facilities and through outpatient referrals covered by Tricare [19]. An exemption for human subjects research was granted for this investigation by the Institutional Review Board at Keller Army Hospital (KACH protocol reference #10/013).

### Population characteristics

Approximately 1.35 million individuals are on active-duty military service each year. The median age in this population is 26 years. Males comprise approximately 88% of the population. The racial distribution is 71% White and 19% Black, and 10% are classified as other. The majority of service members on active-duty are married and serve in the enlisted ranks. All active-duty US military personnel have free and open access to medical care through the Military Health System. In addition to receiving all of their medical treatment through a closed healthcare system, all prospective service members are screened to ensure they meet military medical fitness standards during the accession process. Upon entry into military service, all candidates complete a comprehensive medical evaluation, and those with any significant psychiatric history, including anxiety disorder, acute stress reactions, or PTSD are likely to be disqualified from military service [1, 22].

### Data acquisition and operational definitions

For the current study, the DMSS was queried to identify all incident cases of PTSD among active-duty US service members between 1999 and 2008 by sex, age, race, marital status, branch of military service, and grade or military rank. Specifically, DMSS was queried for ICD-9-CM code 309.81 (prolonged posttraumatic stress disorder). Standard age categories within DMSS were used and included; < 20, 20 to 24, 25 to 29, 30 to 34, 35 to 39, and ≥ 40 years [20]. Race categories included White, Black, and Other, which are also standard categories used within DMSS [20]. Those classified as Other included Hispanic, Asian, Native American and other racial groups based on data contained within the Defense Manpower Data Center Database. Marital status included Married, Single, and Other with the latter category representing those who were divorced or separated. The service categories were Army, Marine Corps, Navy, and Air Force, representing each branch of military service. The categories for military rank included junior enlisted (E1-E4), senior enlisted (E5-E9), junior officer (O1-O4), and senior officer (O5-O9).

Only data from ambulatory visits were used to determine the total number of incident cases with prolonged posttraumatic stress disorder documented as a primary diagnosis. Cases were limited to “first occurrences” to exclude repeat coding for the same diagnosis for all service members during the study period. Similar to previous studies [23, 24], a “first occurrence” was operationally defined so that all incident cases during the study period represented the first documented case of PTSD for each individual from the time they entered military service and excluded all subsequent healthcare visits for PTSD.

### Outcome measures

The incidence rate of PTSD per 1000 person-years at risk during the study period was the primary outcome of interest in this investigation. Precise time-at-risk denominator data (person-time) for incidence rate calculations, which are routinely validated against personnel data contained in the Defense Manpower Data Center Database for quality control purposes, were available through DMSS by strata (e.g., sex, age, race, etc.) for the entire population of interest. During the study period, person-time at risk for PTSD was calculated from the beginning of the study period starting 1 January 1999 until a subject 1) sustained an incident case of PTSD, 2) was separated from military service, or 3) reached the administrative end of the study on December 31st, 2008. For all subjects who joined the military after January 1st, 1999, person-time at risk began accumulating on the date of entry into military service until one of the study end points described above was reached.

### Statistical analyses

The overall unadjusted incidence rate of PTSD, along with the 95% confidence interval (CI), was calculated in aggregate for the entire study period by dividing the total number of incident cases by the total person-years of follow-up and rates were expressed per 1000 person-years. Similarly, unadjusted annual incidence rates were also calculated. Because several demographic and occupational factors were expected to influence the incidence rate of PTSD, a multivariable Poisson regression model was used to analyze the data. Poisson regression **models** the long-stransform of the rate of PTSD as a function of a linear combination of predictor variables and is most appropriate when injury counts are the outcome of interest [25]. We calculated the unadjusted and adjusted incidence rates and rate ratios for all of the demographic (sex, age, race, marital status) and occupational (branch of military service and military rank) factors individually for the entire study period.

Using model-adjusted incidence rates of PTSD, we estimated the average annual percentage change in the rate of PTSD from 1999 through 2002 (baseline period prior to the initiation of combat operations in Iraq) and from 2003 through 2008 (following the initiation of combat operations in Iraq), along with 95% CIs and *p*-values for the observed trends. Because the annual incidence rates of PTSD were relatively stable prior to the initiation of combat operations in Iraq in some instances the aggregate rate from 1999 through 2002 was calculated and used as the baseline rate for subsequent comparisons after the initiation of combat operations. Similar analyses were conducted by sex, age, race, marital status, branch of military service, and military rank. All data were analyzed using SAS software (version 9.2, Cary, NC, US).

## Results

**Fig. 1:**
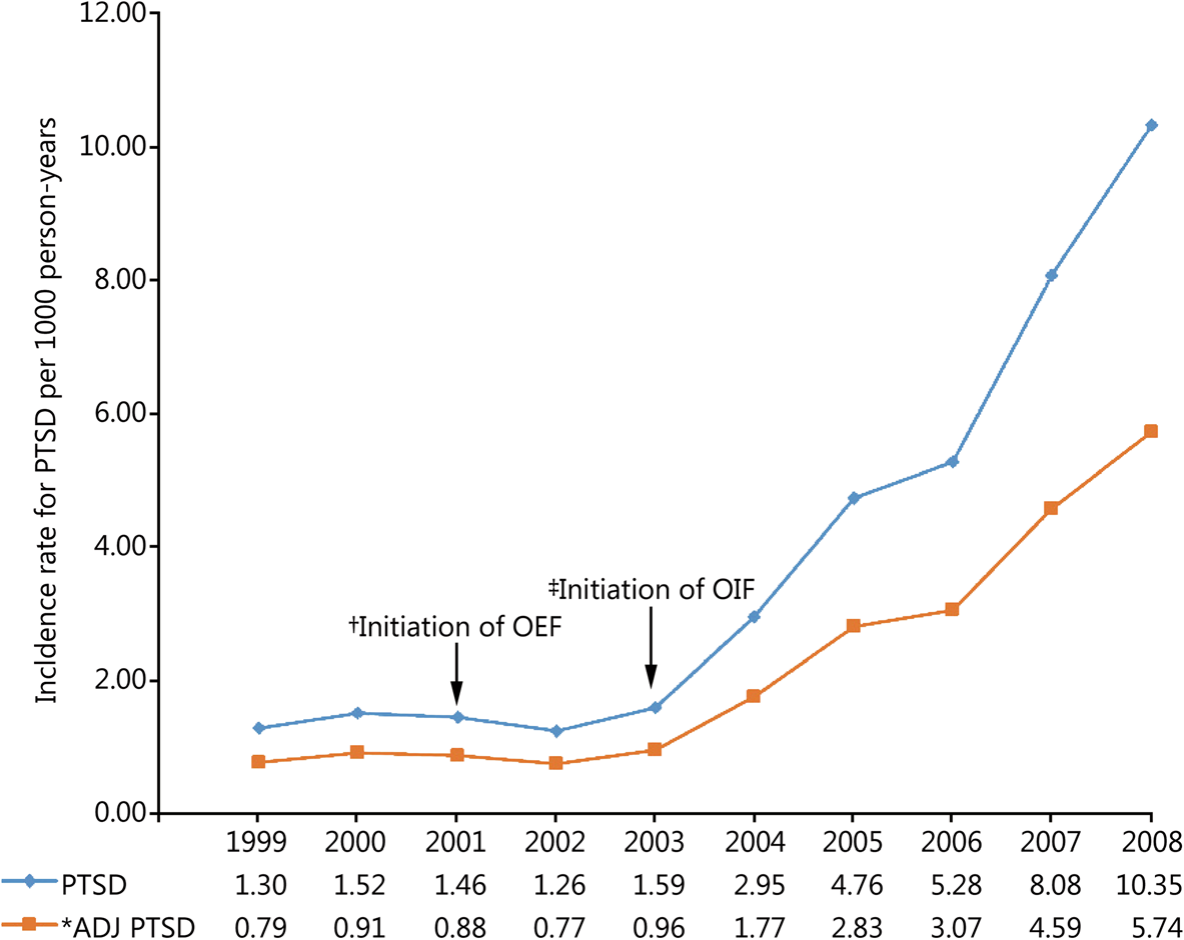
Annual unadjusted and adjusted incidence rates of posttraumatic stress disorder among all active-duty US service members between 1999 and 2008. *.Adjusted for sex, age, race, marital status, rank, and branch of military service; ^†^.Operation Enduring Freedom in Afghanistan, OEF; ^‡^.Operation Iraqi Freedom in Iraq, OIF

**Fig. 2:**
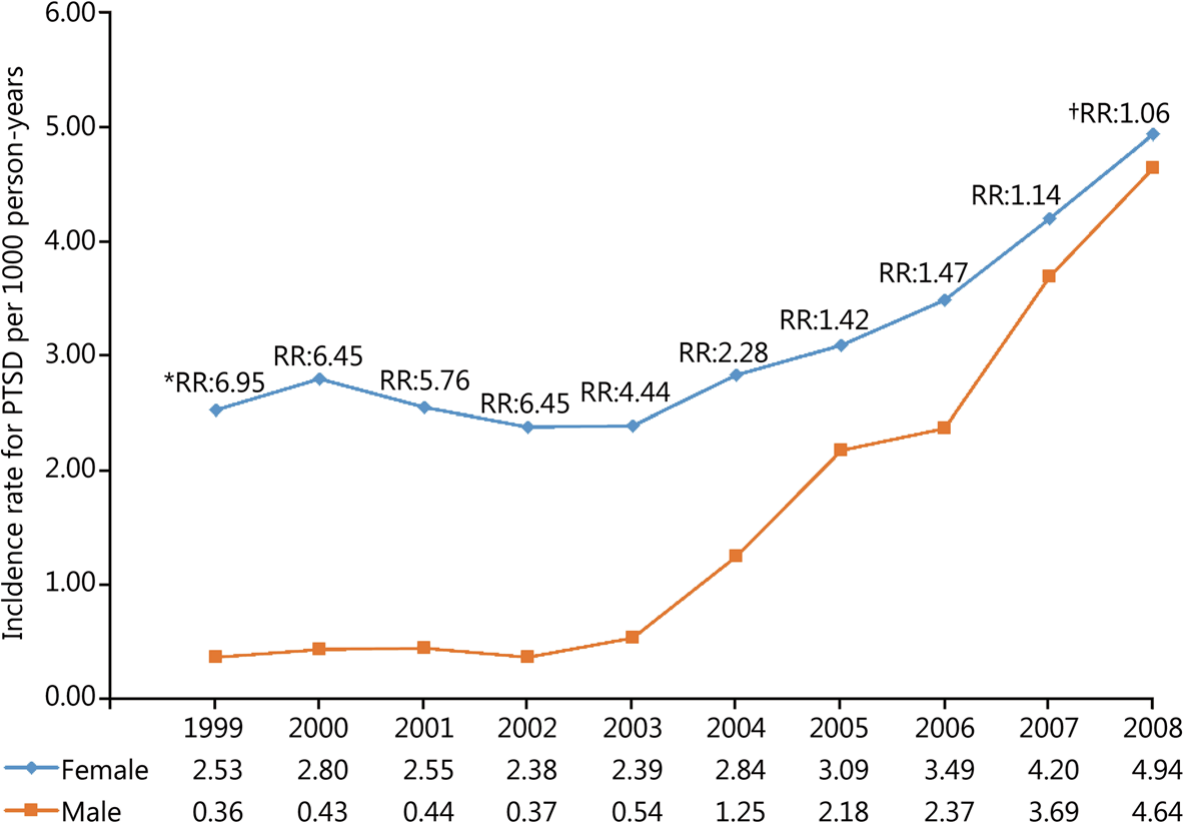
Annual incidence rates of posttraumatic stress disorder by sex among all active-duty US service members between 1999 and 2008. *. Annual incidence rate ratios (RRs) for PTSD between females and males using males as the referent category. All rates and rate ratios are adjusted for age, race, marital status, military rank and branch of military service. All annual rate ratios by sex are statistically significant (*p* < 0.001) except for the year ^†^2008 where there was no gender difference in the incidence rate of PTSD

**Fig. 3:**
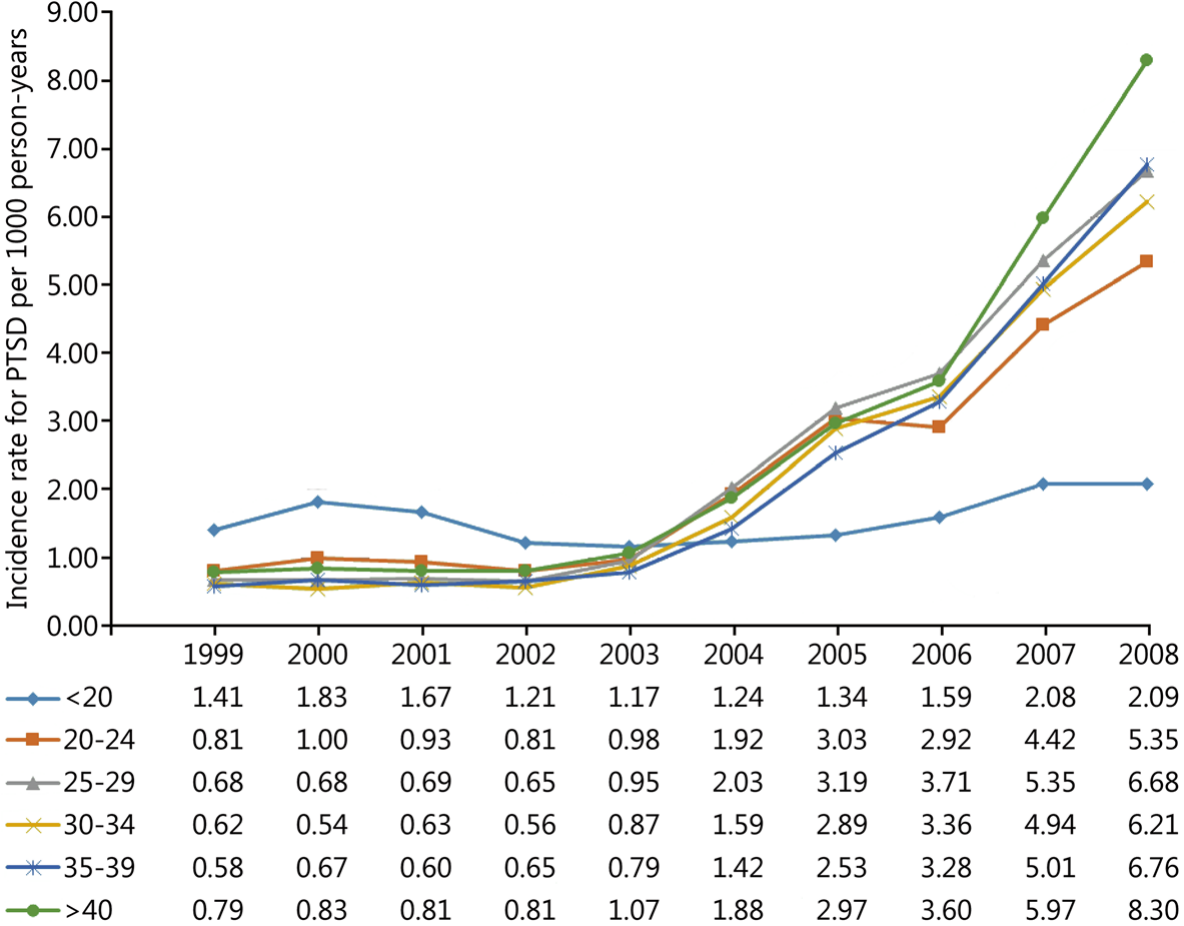
Annual incidence rates of posttraumatic stress disorder by age among all active-duty US service members between 1999 and 2008. All rates are adjusted for sex, race, marital status, military rank and branch of military service

**Fig. 4:**
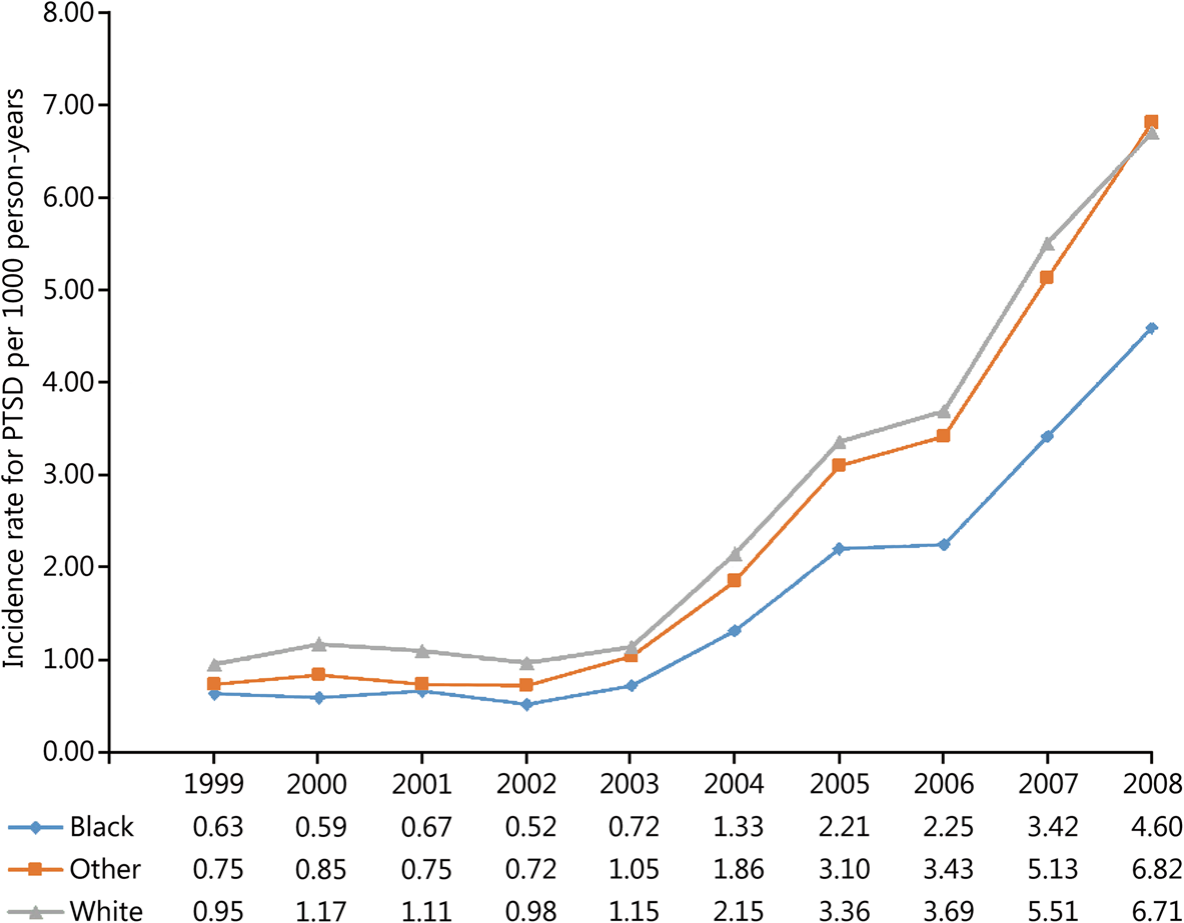
Annual incidence rates of posttraumatic stress disorder by race among all active-duty US service members between 1999 and 2008. All rates are adjusted for sex, age, marital status, military rank and branch of military service

**Fig. 5:**
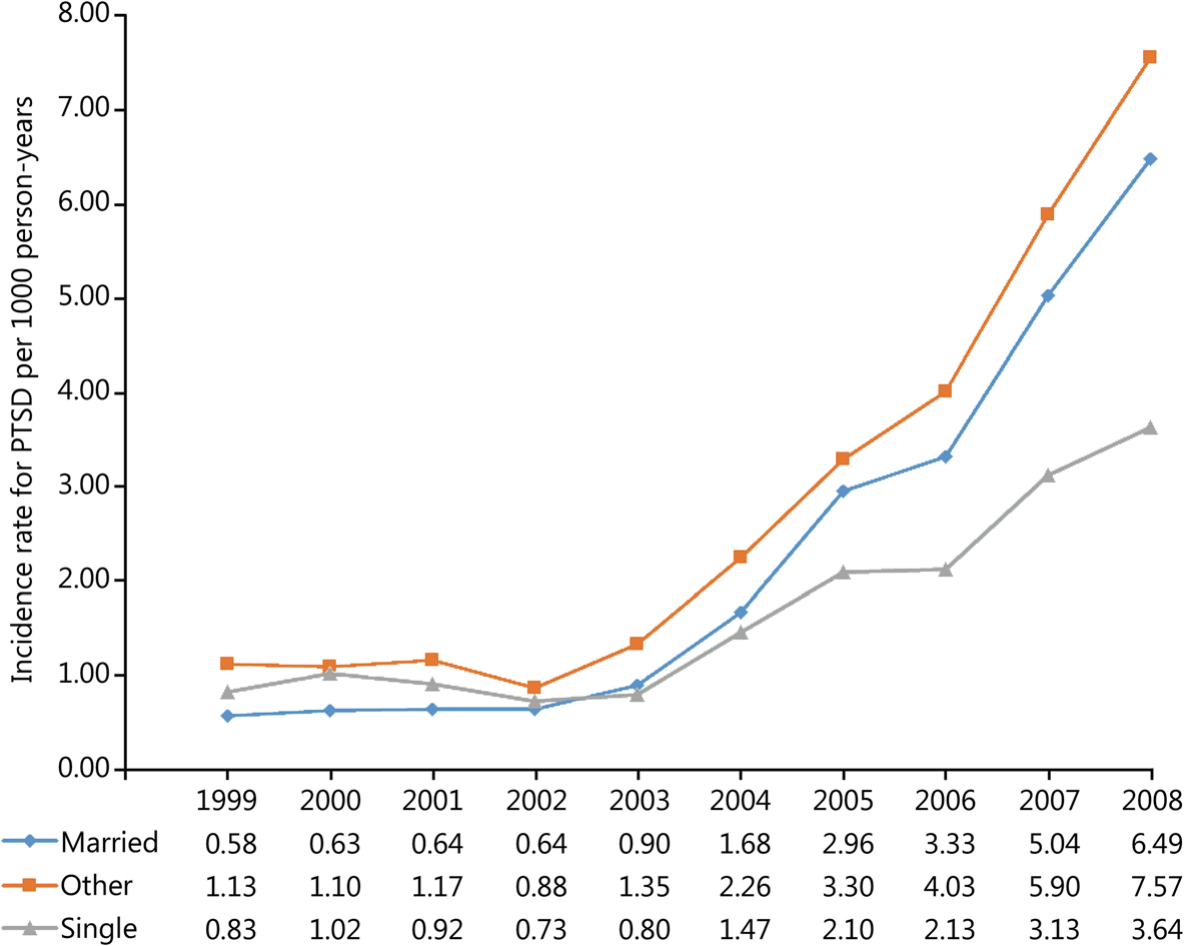
Annual incidence rates of posttraumatic stress disorder by marital status among all active-duty US service members between 1999 and 2008. All rates are adjusted for sex, age, race, military rank and branch of military service

**Fig. 6:**
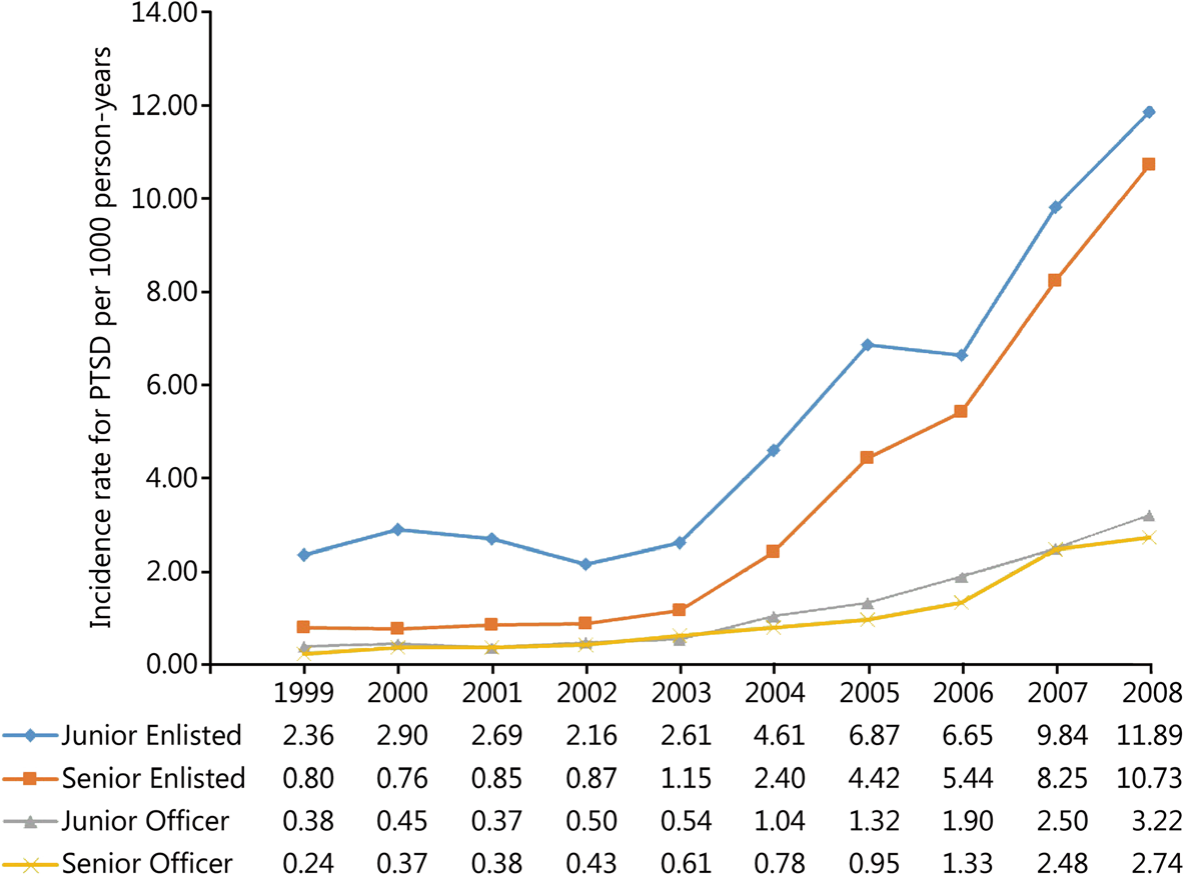
Annual incidence rates of posttraumatic stress disorder by military rank among all active-duty US service members between 1999 and 2008. All rates are adjusted for sex, age, race, marital status and branch of military service

**Fig. 7:**
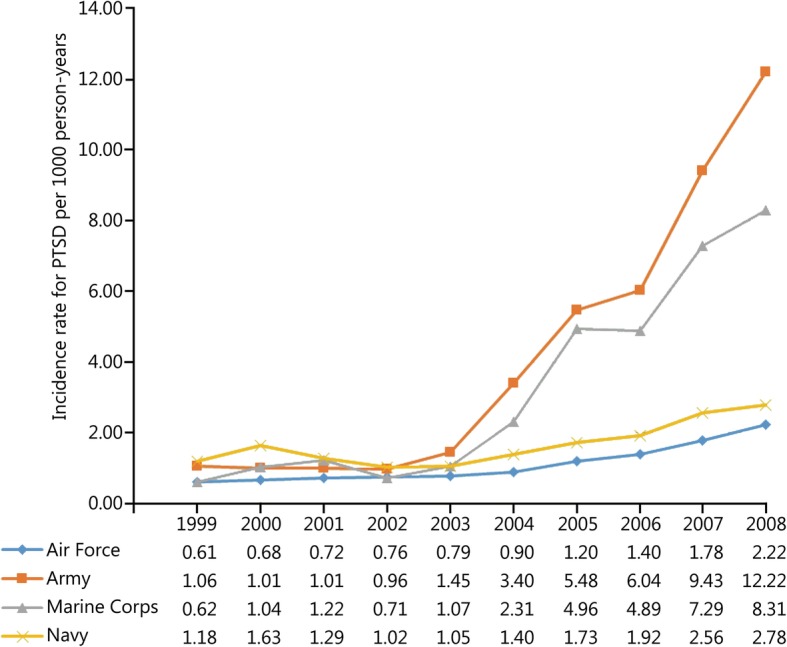
Annual incidence rates of posttraumatic Stress Disorder by branch of military service among all active-duty US service members between 1999 and 2008.All rates are adjusted for sex, age, race, marital status and military rank

During the study period 52,771 incident cases of PTSD and 13,749,746 person-years of follow-up among active-duty US military personnel were documented in DMSS. The overall incidence rate of PTSD in this population was 3.84 (95% CI: 3.81, 3.87) cases per 1000 person-years over the 10-year study period. Trends in the unadjusted and adjusted annual incidence rates of PTSD are presented in Fig. 1. The rate of PTSD was relatively stable prior to the initiation of combat operations in Iraq in March 2003; however, a substantial increase in the rate was observed in subsequent years (Table 1). The average annual increase in the incidence rate of PTSD was 5.02% (95% CI: 1.85, 8.29%) between 1999 and 2002. Following the initiation of combat operations in Iraq the average annual increase in the rate of PTSD was 43.03% (95% CI: 40.55, 45.56%). Compared to the baseline period between 1999 and 2002, the incidence rate of PTSD in 2008 was nearly 7 times higher (RR = 6.85, 95% CI: 6.49, 7.24). After controlling for the other variables in the statistical model, similar trends were observed for the incidence rate of PTSD regardless of sex (Fig. 2), age (Fig. 3), race (Fig. 4), marital status (Fig. 5), military rank (Fig. 6), and branch of military service (Fig. 7), although the magnitude of these trends varied by subgroup.

**Table 1:** Average annual percent change in the incidence rate for PTSD prior to and following the initiation of Combat Operations in Iraq

Item	Baseline 1999–2002			During OIF 2003–2008		
	Annual change (%)	95% CI	*p*	Annual change (%)	95% CI	*p*
All active-duty^a^	5.0	1.9, 8.3	0.002	43.0	40.6, 45.6	< 0.001
Sex^b^						
Female	−1.3	−5.1, 2.6	0.496	15.6	12.5,18.7	< 0.001
Male	10.4	6.4, 14.4	< 0.001	53.8	50.9,56.8	< 0.001
Age^b^						
< 20	−4.7	−11.5, 2.6	0.205	12.4	6.3, 18.8	< 0.001
20–24	4.8	0.1, 9.7	0.045	40.4	36.8, 44.1	< 0.001
25–29	8.6	1.6, 16.1	0.015	47.8	42.5, 53.3	< 0.001
30–34	9.1	0.0, 19.0	0.049	48.1	41.2, 55.4	< 0.001
35–40	8.0	−2.3, 19.3	0.133	53.7	45.3, 62.6	< 0.001
> 40	8.0	−3.4, 20.8	0.178	50.7	42.0, 59.8	< 0.001
Race^b^						
Black	3.3	−3.9, 11.1	0.374	44.8	38.6, 51.2	< 0.001
Other	8.9	−0.3, 18.9	0.059	45.3	38.6, 52.4	< 0.001
White	4.8	1.0, 8.7	0.012	42.3	39.4, 45.3	< 0.001
Marital Status^b^						
Married	11.5	6.6, 16.5	< 0.001	48.6	45.1, 52.2	< 0.001
Other	4.5	−7.4, 17.9	0.479	41.3	31.7, 51.5	< 0.001
Single	−0.9	−4.9, 3.3	0.669	35.4	32.0, 38.9	< 0.001
Rank^b^						
E1-E4	2.6	−1.0, 6.3	0.156	35.4	32.5, 38.3	< 0.001
E5-E9	9.4	3.3, 15.9	0.002	56.3	51.6, 61.1	< 0.001
O1-O3	8.9	−7.5, 28.3	0.305	42.9	30.6, 56.3	< 0.001
O4-O9	26.8	1.6, 58.4	0.036	35.0	21.8, 49.6	< 0.001
Service^b^						
Army	8.2	3.7, 12.8	< 0.001	53.2	49.8, 56.7	< 0.001
Marines	14.5	5.0, 24.9	0.002	50.7	44.2, 57.5	< 0.001
Navy	−3.0	−7.8, 2.0	0.228	21.6	17.4, 25.9	< 0.001
Air Force	6.8	0.3, 13.6	0.039	23.0	18.4, 27.9	< 0.001

^a^ Adjusted for sex, age, race, marital status, military rank, and branch of military service; ^b^ Adjusted for all other variables in the table

The magnitude of the observed increase in the rate of PTSD was much greater for males than females. From 2003 through 2008, the average annual increase in the incidence rate of PTSD was 53.8% among males compared to 15.6% for females. Overall, after adjusting for the other variables, the incidence rate of PTSD among female service members was nearly twice as high when compared to males during the entire study period (Table 2). However, there was a significant time-by-sex interaction for the incidence rate of PTSD (*p* < 0.001) during the study period. Females experienced much higher incidence rates for PTSD prior to the initiation of combat operations in Iraq, but this disparity was eliminated by 2008 (Fig. 2).

**Table 2:** Incidence rates and rate ratios for PTSD by demographic and occupational factors among active-duty US service members between 1999 and 2008

Item	Cases	%	Person-years	PTSD rate (cases)^a^	95% CI	Unadjusted rate ratio (95% CI)		Adjusted rate^b^ ratio (95% CI)	
Sex									
Female	11,396	21.58	1,966,227	5.80	5.44, 6.17	1.65	1.54, 1.77	1.92	1.84, 2.00
Male	41,410	78.42	11,783,519	3.51	3.40, 3.63	(ref)	–	(ref)	–
Age									
< 20	3565	6.75	1,103,833	3.23	2.89, 3.61	(ref)	–	(ref)	–
20–24	20,745	39.29	4,540,468	4.57	4.36, 4.79	1.41	1.25, 1.60	1.36	1.27, 1.46
25–29	12,645	23.95	2,885,197	4.38	4.13, 4.65	1.36	1.20, 1.54	1.52	1.41, 1.65
30–34	6526	12.36	2,021,167	3.23	2.97, 3.51	1.00	0.87, 1.15	1.37	1.25, 1.50
35–39	5116	9.69	1,800,101	2.84	2.59, 3.12	0.88	0.76, 1.02	1.37	1.24, 1.52
> 39	4209	7.97	1,398,980	3.01	2.71, 3.33	0.93	0.80, 1.08	1.68	1.51, 1.87
Race									
Black	7966	15.09	2,654,476	3.00	2.79, 3.23	(ref)	–	(ref)	–
Other	6772	12.82	1,708,460	3.96	3.66, 4.30	1.32	1.18, 1.47	1.45	1.36, 1.54
White	38,068	72.09	9,386,810	4.06	3.92, 4.20	1.35	1.25, 1.47	1.58	1.51, 1.66
Marital Status									
Married	29,318	55.52	7,403,616	3.96	3.81, 4.11	1.13	1.06, 1.20	1.38	1.32, 1.44
Other	3172	6.01	568,459	5.58	4.97, 6.27	1.59	1.40, 1.80	1.72	1.59, 1.86
Single	20,316	38.47	5,777,671	3.52	3.36, 3.68	(ref)		(ref)	
Rank									
E1-E4	29,302	55.49	6,044,647	4.85	4.67, 5.03	3.90	3.19, 4.77	4.93	4.31, 5.63
E5-E9	20,640	39.09	5,501,415	3.75	3.59, 3.92	3.02	2.47, 3.70	3.42	3.02, 3.89
O1-O3	1791	3.39	1,339,516	1.34	1.15, 1.56	1.08	0.84, 1.38	1.17	1.00, 1.36
O4-O9	1073	2.03	864,168	1.24	1.02, 1.51	(ref)	–	(ref)	–
Service									
Air Force	5747	10.88	3,502,310	1.64	1.52, 1.77	(ref)	–	(ref)	–
Army	30,572	57.89	4,904,786	6.23	6.04, 6.44	3.80	3.50, 4.12	3.80	3.59, 4.02
Marines	8440	15.98	1,776,267	4.75	4.47, 5.05	2.90	2.63, 3.19	2.92	2.73, 3.12
Navy	8047	15.24	3,566,383	2.26	2.12, 2.40	1.38	1.25, 1.52	1.51	1.41, 1.61

^a^ Unadjusted incidence rate of PTSD per 1000 person-years; ^b^ Adjusted for all other variables in the table; (ref). Reference category for incidence rate ratio comparisons per variable

Incidence rates, unadjusted and adjusted rate ratios, and 95% CIs for each demographic and occupational factor during the entire study period are presented in Table 2. During the entire study period, unadjusted incidence rates for PTSD were highest among service members in their third decade of life; however, after adjusting for the other variables in the model, the incidence rates of PTSD were highest among service members who were 40 years of age and older, followed by those in the 25–29 year-old age group. Overall, service members in the Black racial category experienced the lowest rate of PTSD during the entire study period. Compared to those in the Black racial group, the incidence rates of PTSD among service members in the Other and White racial categories were 45 and 58% higher, respectively, after adjustment. Service members who were single experienced the lowest rate of PTSD during the entire study period after controlling for the other variables in the model. Compared to single service members, the incidence rate of PTSD was 38% higher among married service members and 72% higher among service members in the Other category for marital status. Senior officers experienced the lowest incidence rate of PTSD during the entire study period, followed by junior officers, senior enlisted, and junior enlisted service members. Compared to senior officers, the incidence rate of PTSD among senior and junior enlisted service members was 3.42 and 4.93 times higher, respectively, after adjusting for the other factors in the model. Finally, during the entire study period, the incidence rate of PTSD was highest among those serving in the Army, followed by those serving in the Marine Corps, Navy, and Air Force. Compared to those serving in the Air Force, the rate of PTSD was 51% higher among those serving in the Navy, 2.92 times higher among those in the Marine Corps, and 3.8 times higher among those in the Army after adjustment for other variables in the model.

## Discussion

This is the first study to examine trends in the annual incidence rate of PTSD before and subsequent to the initiation of combat operations in Iraq and Afghanistan among active-duty military personnel within all four branches of military service. To our knowledge, no similar study examining trends in the incidence rate of PTSD before and after the initiation of combat operations has been conducted in relation to prior US military engagements (e.g., Vietnam, Korea, etc.). While recent studies have reported baseline rates for mental health disorders among military personnel, none have reported baseline rates for PTSD specifically [1, 2]. Although OEF was initiated in late 2001, the results of the present study indicate that incidence rates of PTSD were relatively stable prior to the initiation of OIF in 2003 and that there was a significant upward trend in the annual rate in subsequent years. Previous studies have reported that both exposure to combat and the prevalence of PTSD were significantly higher among active-duty service members deployed to Iraq when compared to those that served in Afghanistan during this time period [26], which may partially explain why the initiation of OEF had little impact on the incidence rate of PTSD in the current study. The increase in the annual rate of PTSD following the initiation of OIF was observed regardless of sex, age, race, marital status, military rank, and branch of military service with few exceptions in the current study; however, the magnitude of the observed increases varied by demographic and occupational subgroup. The findings of the current study have significant policy implications.

In the only other population-based study to report incidence rates of PTSD among military personnel, Smith et al. [15] reported cumulative incidence rates ranging from 10 to 13 cases per 1000 person-years. They used the posttraumatic stress disorder checklist (PCL) to identify incident cases of PTSD in the Millennium Cohort Study population, which oversampled female, previously deployed, and Reserve/National Guard personnel by design. Despite the fact that these two studies used different case definitions for PTSD and that the Millennium Cohort subjects may not be representative of the military population in general, the incidence rates reported by Smith et al. [15] are comparable to the incidence rate reported in the final year of the current study. We observed an overall incidence rate of nearly 4 cases of PTSD per 1000 person-years during the entire study period; however, the annual incidence rate in the final year of the current study was 10.35 cases per 1000 person-years. Similarly, the results for the association between sex, military rank, and marital status, and the incidence rate of PTSD were also consistent between the present study and the study by Smith et al. [15].

The observed increase in the incidence rate of PTSD following the initiation of OIF is likely due to the substantial increase in combat exposure and potentially the stressors associated with deployment experienced by active-duty military populations during this time period. Combat exposure has recently been examined in several studies [6, 8, 15, 26–30] and recent review articles have identified combat exposure as the most important factor associated with the prevalence and incidence of PTSD among military service members [9, 10]. Smith et al. [15] reported that as many as 76% of the incident cases of PTSD may be attributed to combat exposure among deployed soldiers; however, they also noted that deployment alone may be insufficient to assess combat exposure. The specific types, severity, and cumulative duration of combat exposure may also influence the incidence and prevalence of PTSD [15, 31, 32]. The significant increases in the annual incidence rate of PTSD observed in the current study may also reflect the cumulative effects of combat exposure over time due to the number of multiple deployments to Iraq and Afghanistan within this population during the study period [31, 32]. A recent study reported that male Marines were twice as likely to experience PTSD following multiple deployments to Iraq and Afghanistan when compared to service members with only one deployment [30]. Furthermore, recent reports from the United States Army Medical Command suggest that personnel with 3 or more deployments are at even greater risk of mental health problems [31, 32]. Although the specific types and cumulative effects of combat exposure were not assessed directly in the current study, we were able to evaluate annual incidence rates during a baseline period prior to and a follow-up period subsequent to the initiation of combat operations in Iraq and Afghanistan. The results of the current study suggest that incidence rates increased substantially after the initiation of combat operations in Iraq, likely due to the increased intensity and cumulative effects of combat exposure in this population.

### Demographic factors

The adjusted incidence rate of PTSD in the current study was nearly twice as high among active-duty female service members when compared to males. This finding is consistent with the results observed by Smith et al. [15], who reported that the incidence of PTSD was 1.7 to 2.0 times higher among female service members when compared to males. Numerous other studies have consistently reported that females are approximately twice as likely to be diagnosed with PTSD when compared to males in the general population [33, 34].

When the annual incidence rates of PTSD by sex were examined over time in the current study, a significant sex-by-time interaction was observed. The incidence rate among female service members was 5.75–6.95 times as high as the rate among males during the baseline period prior to OIF. This is consistent with the existing literature that has reported higher rates of baseline and predeployment mental health disorders among female service members when compared to males [1, 35, 36]. Although baseline rates prior to OIF were significantly higher among female service members, by the final year of our study there was no difference in the rate of PTSD by sex. This interaction is likely due to differences in combat exposure between males and females. Females were not permitted to serve in traditional combat units, and previous studies have reported that males were much more likely to be deployed in support of OIF and OEF [15]. Furthermore, the types of combat exposure experienced by males and females may differ. For example, Hoge et al. [37] reported that women were more likely to experience combat exposures that were associated with the aftermath of war (e.g., handling human remains) while males were more likely to experience direct combat exposures (e.g., being in fire fights or directing fire at the enemy). As a result, it appears that controlling for the specific types of combat exposure, including the cumulative effects of combat exposure, as well as predeployment mental health status may be important in future studies examining sex-specific factors associated with PTSD in active-duty populations.

The incidence rate of PTSD increased substantially following the initiation of OIF in all age groups in the current study, with the notable exception of those in the less than 20 year age group. This may be because many service members in this age group are completing initial entry level and advanced training prior to deployment, and as a result do not experience the same combat exposures as the other groups. Because we were unable to directly assess combat exposure in the current study, we could not control for this factor in our analysis. In a recent review article, Ramchand et al. [9] reported that the association between age and PTSD was not found to be significant in bivariate analyses or multivariate models or lost significance after adjustment in the five studies that examined this relationship. In the current study, age was associated with the incidence rate of PTSD. The unadjusted rates were highest in those who were 20–29 years of age; however, after adjustment, the rates were highest in the oldest age group followed by those who were 25–29 years of age. Because we did not directly control for combat exposure in our analysis, it is possible that those in the oldest age group had the greatest cumulative amount of combat experience during the study period. This is consistent with other reports indicating that cumulative combat exposure places older enlisted service members at greater risk for PTSD [31, 32]. Although we observed a significant association between age and the incidence rate of PTSD, there was no clear pattern to this relationship.

Overall, incidence rates of PTSD were highest among divorced service members in the current study, followed by service members who were married and single. Smith et al. [15] reported similar findings, noting that the incidence of PTSD was significantly higher among divorced service members in the Air Force, Navy and Coast Guard, and Marine Corps. A recent study suggested that social support was an important factor associated with postdeployment PTSD; therefore, it seems plausible that divorced service members may have inadequate social support following deployment [38]. In contrast, Smith et al. [15] reported no difference in the incidence of PTSD between single and married service members; however, in the present study the incidence rate of PTSD was 38% higher among married service members when compared to those who were single. Although some studies suggest that social support may be important in preventing and mitigating the effects of PTSD, other studies suggest that military deployments and combat exposure may lead to increased stress and negatively impact marital relations following deployment [39]. Recently, Milliken et al. [40] reported that military service members returning from deployments reported a four-fold increase in interpersonal conflict during the subsequent 6 months. As a result, they suggested that military spouses may play an important role in encouraging service members to seek care for mental health issues following deployments. This may, in part, explain why higher incidence rates of PTSD were observed among married service members in comparison to those who were single. Conversely, it is plausible that many single service members, in the absence of the social support associated with marriage, do not seek care for mental health conditions.

The association between racial group affiliation and the risk of PTSD has been variably reported in the literature. Several studies in Vietnam veteran populations have reported that Black and Hispanic racial groups are at an increased risk for PTSD when compared to White veterans [41–44] and several possible factors were proposed to explain these differences; however, a compelling or comprehensive theory to explain these group differences has yet to emerge [41]. Dlugosz et al. [45] reported that the rate of hospitalization for adjustment disorders (including PTSD) in military personnel following the first Persian Gulf War was highest in the White racial group, followed by those in the Other and Black groups. This is consistent with the results observed in the current study. In contrast to the current study, no clear pattern was observed in relation to differences in the incidence of PTSD by racial group within military service members deployed to Iraq and Afghanistan in the Millennium Cohort study [15]. As a result, the association between race and the incidence of PTSD in military service members remains unclear and further research in this area is needed.

The fact that the observed annual incidence rates of PTSD were consistently lower in the Black racial group in the current study is noteworthy. Because all active-duty US military service members have free and open access to health care through the Military Health System, it is unlikely that the observed difference in the incidence rate of PTSD is due to disparities in access to care; however, differences in care-seeking behavior may be a plausible explanation. Further study is needed to better identify the factors that mediate the relationship between race and the incidence rate of PTSD in active-duty service members.

### Occupational factors

We observed a significant association between occupational factors such as military rank and branch of military service and the incidence rate of PTSD in the current study. Although significant increases in the rate of PTSD were observed subsequent to the initiation of OIF regardless of rank, the magnitude of the change was most notable among enlisted service members, both junior and senior. Previous studies have consistently demonstrated that junior and senior enlisted service members are at the greatest risk for PTSD [9, 15, 30]. Despite the overall lower incidence rates for PTSD among officers in the current study, some findings suggest that persistent self-reported symptoms of PTSD are proportionately higher among officers [15]. The higher rates of PTSD among junior and senior enlisted service members in the current study are likely associated with the intensity, duration, and types of combat experienced within these groups [26]. Similar to the results for rank, significant increases in the rate of PTSD were observed subsequent to the initiation of OIF regardless of branch of military service; however the magnitude of change was most notable among those serving in the Army and Marine Corps. Hoge et al. [26] reported that nearly 40% of all active-duty Army and Marine Corps personnel had been deployed in support of OIF and OEF by July of 2004 and by the end of the current study many service members in the Army and Marine Corps had served multiple deployments in these theaters of operation [30–32]. Recent studies have also reported that service in the Army and Marine Corps is associated with a significantly higher risk of PTSD [46]. As a result, the observed differences in the incidence rate of PTSD over time by branch of military service are also likely due to differences in the type, intensity, and cumulative duration of combat exposure experienced by these groups.

### Limitations

The current study had notable limitations that should be considered when interpreting the findings, and we have highlighted many of these in similar studies previously [17, 18]. We did not directly measure combat exposure during the current study; however, since we examined data for the entire active-duty population, we can indirectly estimate when combat exposure increased in the population from records. While it is likely that the observed increase in the incidence of PTSD following the initiation of combat operations in Iraq and Afghanistan is associated with the increase in combat exposure during the study period, we cannot rule out that the observed increase was due to other deployment related stressors during the study period or index trauma that was experienced prior to the increased operational tempo but not reported until a later date. We examined the incidence of PTSD among active-duty military service members. Therefore, the observed results may not be applicable to Reserve or National Guard service members, who may experience a higher incidence and prevalence of PTSD according to previous studies [4, 9, 10, 47]. Another limitation of the current study is that we relied on physician diagnosed cases of prolonged PTSD to identify incident cases. Previous studies have suggested that this approach may underestimate the true incidence of PTSD in military populations as many service members may not seek care due to perceived stigma and other barriers [26]. Furthermore, it is possible that some active-duty service members may have delayed reporting PTSD symptom onset until after the study period, which may also contribute to underestimating the true incidence of PTSD in the population. The current study utilized administrative data from the DMSS to estimate incidence rates for PTSD over a 10-year period. The limitations of this approach have been discussed previously [16, 24, 48]. The quality of the administrative data utilized in the current study is dependent on the completeness, validity, consistency, timeliness and accuracy of the data contained within the DMSS [20]. Coding errors associated with incident case diagnoses cannot be ruled out when using large administrative databases for epidemiological research purposes. Misclassification of the outcome of interest introduces the potential for information bias, which may have also resulted in under estimation of the true incidence of PTSD in the current study; however, the likelihood of any differential misclassification is limited because these data are representative of multiple providers throughout the Military Health System who were unaware of whether participants were exposed to the factors of interest [25]. Because we included only incident cases seen in ambulatory settings, it is possible that cases associated with injury and comorbid conditions requiring hospitalization or more severe cases of PTSD that were not treated in ambulatory clinics may have been missed. Another limitation is the longitudinal nature of the current study, as changes in the awareness and documentation requirements for PTSD among health care providers may have changed during the study period and this may have accounted, in part, for the observed increases in the incidence rate of PTSD. A final limitation is that the data contained within DMSS may not capture cases of PTSD or acute stress reactions that are managed in theater; however, it is likely that the majority of cases of prolonged PTSD would be identified during postdeployment health appraisals or when service members with prolonged PTSD were medically evacuated from theatre.

## Conclusions

The initiation of combat operations in Iraq and Afghanistan resulted in a substantial increase in the incidence rate of PTSD among active-duty service members. Significant increases in the rate of PTSD were observed following the initiation of OIF regardless of sex, age, race, marital status, military rank, and branch of military service. The planning of future combat operations, particularly prolonged large scale missions, should better anticipate the burden of mental health conditions such as PTSD and ensure that adequate mental health resources are available for military service members and their families from the outset. Similarly, effective primary and secondary prevention strategies targeting the most vulnerable groups noted above are needed to mitigate the impacts of PTSD subsequent to the initiation of combat operations in the future.

## Abbreviations

CI: Confidence interval
DMSS: Defense Medical Surveillance System
ICD-9-CM: International Classification of Diseases, Ninth Revision, Clinical Modification
OEF: Operation Enduring Freedom (Afghanistan)
OIF: Operation Iraqi Freedom
PTSD: Posttraumatic stress disorder
RR: Rate ratio
